# ID 83 - Avaliação de Tecnologias em Saúde em Mato Grosso: Estratégias Utilizadas no Processo de Institucionalização do Nats-SES-MT

**DOI:** 10.5327/2237-9622.2025.v34s1.83

**Published:** 2025-11-25

**Authors:** Kelli Carneiro de Freitas Nakata, Helder Cássio de Oliveira, Graciane Catarina Batista Magalhães, Lucí Emilia Grzybowski de Oliveira, Marcelo Maia Pinheiro, Elton Hugo Maia Teixeira, Maria do Carmo Souza, Ternize Mariana Guenkka, Gilson Yugi Nakata, Zenóbia Quinderé Barreto

## Abstract

**Introdução:**

Desde a instituição da Comissão Permanente de Farmácia e Terapêutica (CPFT) por meio da Portaria n.º 028/2014/GBSES de 2 de março de 2014, Mato Grosso tem realizado esforços significativos para integrar a avaliação de tecnologias em saúde (ATS) na gestão do Sistema Único de Saúde (SUS). Transformada em Núcleo de Avaliação de Tecnologias em Saúde (Nats) em 2017 e vinculada à Rede Brasileira de Avaliação de Tecnologias em Saúde (Rebrats), a CPFT/NATS tem adotado uma abordagem multiprofissional para melhorar as políticas de saúde por meio de uma série de iniciativas inovadoras.

Essas iniciativas incluem a elaboração de pareceres técnicos-científicos para informar e orientar decisões políticas e administrativas, a produção de protocolos de uso de medicamentos e a Relação Estadual de Medicamentos (Resme). Adicionalmente, o Nats tem investido na produção de materiais educativos como vídeos e podcasts para facilitar a compreensão e a disseminação de conceitos de ATS para a comunidade e gestores de saúde. Destaca-se também a coordenação de um programa de pós-graduação em ATS, realizado em parceria com o Nats-HUJM e a Escola de Saúde Pública da SES-MT, ampliando o alcance e a profundidade da capacitação em ATS no estado.

Uma novidade importante nas estratégias de divulgação da ATS foi a criação de plataformas digitais, incluindo uma conta no Instagram e um grupo no WhatsApp. Estes canais têm desempenhado um papel crucial na disseminação rápida e eficaz de informações sobre ATS para um público mais amplo, incluindo profissionais de saúde e o público geral. As mídias sociais permitem interações diretas e em tempo real, facilitando a disseminação de informações atualizadas e a promoção de eventos e iniciativas educacionais.

O engajamento direto com os diversos setores da SES-MT por meio de visitas locais tem sido crucial para a promoção da compreensão e apoio à ATS.

Essas ações educativas e de advocacy são complementadas pela publicação regular de boletins informativos que mantêm os profissionais atualizados sobre as últimas tendências e evidências em ATS.

O sucesso dessas iniciativas é evidenciado pela formalização da CPFT/Nats, concretizada por sua recente inclusão no organograma da Secretaria Estadual de Saúde, conforme estabelecido pelo Decreto n.º 766, de 04 de março de 2024. Este reconhecimento institucional não só valida o trabalho realizado como também assegura a sustentabilidade e a continuidade da integração da ATS nas políticas de saúde do estado. A avaliação de tecnologia em saúde agora é uma parte integral do Plano Estadual de Saúde 2024/2027, consolidando o papel da ATS na melhoria da gestão de saúde em Mato Grosso.

Este conjunto de ações reflete um modelo robusto de como a ATS pode ser democratizada e institucionalizada dentro de uma gestão de saúde estadual, proporcionando um exemplo valioso para outras regiões e estados do Brasil. A apresentação destas iniciativas no V Congresso da Rebrats será uma oportunidade para destacar os benefícios da integração da ATS no SUS, demonstrando a aplicabilidade e os impactos positivos dessas estratégias no sistema de saúde.

**Método:**

Apresentação será por meio de eslaides e em seguida será demonstrado onde encontrar todas as informações no site da CPFT/Nats-HUJM. O roteiro da apresentação será da seguinte forma:

O Roteiro da apresentação seguirá os seguintes passos:

1. Introdução (1 minuto): apresentação do contexto e da evolução da CPFT para Nats, vinculação à Rebrats e impacto na gestão de saúde em Mato Grosso.
2. Iniciativas implementadas (3 minutos):
  - Produtos de ATS: como pareceres técnicos-científicos, MHT, impacto orçamentário e protocolo de dispensação.
  - Materiais Educativos: mostrar os materiais elaborados no site da CPFT/Nats
  - Visita in loco: explicar como foi realizado.
  - Capacitação: informações sobre o curso de especialização em ATS, o primeiro do estado, oferecido gratuitamente para funcionários públicos.
  - Institucionalização da CPFT/Nats: informar que esta comissão foi inserida no organograma da SES-MT e práticas de ATS inseridas no plano estadual de saúde.
3. Conclusão (1 minuto): reflexões sobre o sucesso da institucionalização da CPFT/Nats na estrutura da SES, inclusão no Plano Estadual de Saúde 2024-2027, e perspectivas futuras para a democratização da ATS em Mato Grosso.
  - Equipamentos necessários: uso de um monitor e datashow para apresentação de vídeos e eslaides, além de equipamento de som para a exibição de trechos de podcasts durante a apresentação e internet para apresentação do site da CPFT/Nats-SES

